# Unlocking high-voltage anode-free sodium–sulfur batteries

**DOI:** 10.1093/nsr/nwag180

**Published:** 2026-03-19

**Authors:** Yongwen Ren, Bao-Lian Su

**Affiliations:** Laboratory of Inorganic Materials Chemistry (CMI), Namur Institute of Structured Matter (NISM), University of Namur, Belgium; Laboratory of Inorganic Materials Chemistry (CMI), Namur Institute of Structured Matter (NISM), University of Namur, Belgium; State Key Laboratory of Advanced Technology for Materials Synthesis and Processing, Wuhan University of Technology, China

Ideal energy storage technologies should be efficient, safe, and cost-effective [1–3]. Room-temperature sodium–sulfur (Na–S) batteries that couple the earth abundance of sodium with the highly tunable reactivity of sulfur have been regarded as a promising candidate for large-scale energy storage [4–6]. However, their practical implementation has been constrained by the intrinsically low discharge voltages (<1.6 V) and the reliance on a substantial excess of sodium metal at the anode [4]. Overcoming these long-standing limitations demands not only incremental optimization, but also a fundamental rethinking of both sulfur redox chemistry and battery architecture.

**Figure 1.**
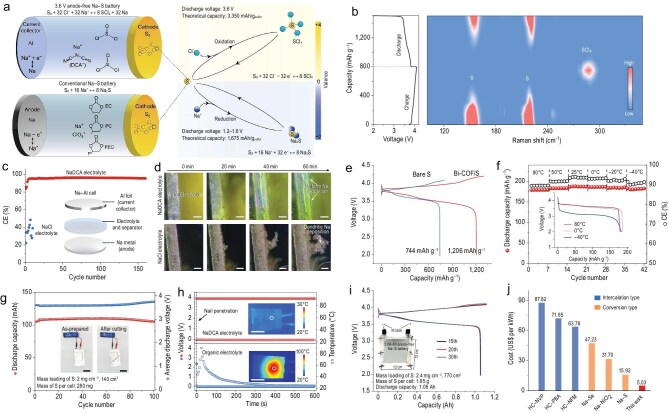
(a) Schematic illustration for the anode-free Na–S battery (involving S/SCl_4_ redox reaction) and conventional Na–S batteries (based on S/Na_2_S redox chemistry). (b) *In situ* Raman spectra of the S cathode in an anode-free Na–S battery at the first cycle. (c) Cycling performance of Na–Al cells using NaDCA and NaCl electrolytes at 1  mA  cm^−2^ and 1 mAh  cm^−2^. The inset illustrates the Na–Al cell. (d) *In situ* optical images of Na plating on Al foil in an Na–Al cell. The current density and plating capacity are 1  mA  cm^−2^ and 1  mAh  cm^−2^, respectively. Scale bars, 500  μm. (e) Galvanostatic charge–discharge curves of anode-free Na–S batteries using bare S and Bi-COF/S cathodes at a current density of 500 mA g^−1^. (f) Discharge capacities of high-voltage anode-free Na–S battery at different temperatures with a charge capacity of 200  mAh g^−1^. The inset shows the representative galvanostatic discharge curves at 80, 0, and −40°C. (g) Cycling performance of a 108-mAh anode-free Na–S pouch cell at a current density of 1200 mA g^−1^ (2.40 mA cm^−2^). The insets show photographs of an anode-free Na–S pouch cell powering a light-emitting diode screen before and after being cut. (h) Variations of temperature and voltage of the anode-free Na–S pouch cell compared with a conventional Na–S pouch cell during nail penetration tests. (i) Galvanostatic charge–discharge curves of a 1.06-Ah high-voltage anode-free Na–S battery at a current density of 500 mA g^−1^. The inset shows the photograph of the battery. (j) Comparison of the unit price of the high-voltage anode-free Na–S battery and some representative rechargeable Na batteries. HC, NVP, PBA, and NFM represent hard carbon, Na_3_V_2_(PO_4_)_3_, Na_2_MnFe(CN)_6_, and NaNi_1/3_Fe_1/3_Mn_1/3_O_2_, respectively. Adapted with permission from ref. [7].

Writing in *Nature*, Sun *et al.* report a conceptual breakthrough on high-voltage, anode-free Na–S batteries, transforming the reaction chemistry and battery architecture [7]. It features a high-valence S^0^/S^4+^ cathode chemistry that yields a discharge voltage of 3.6 V while eliminating the need for preloaded Na metal (Fig. 1a). The innovation of this work lies in the introduction of the sodium dicyanamide (NaDCA) salt into a non-flammable chloroaluminate electrolyte. This additive plays a dual role: facilitating the reversible conversion between S_8_ and SCl_4_, and promoting the reversibility of Na plating and stripping at the anode through forming a nitrogen-rich solid-electrolyte interphase layer. These synergistic effects give rise to an unconventional Na–S chemistry that combines high operating voltage with excellent reversibility, laying the foundation for the exceptional electrochemical performance reported.

*In situ* Raman spectroscopy reveals the reversible evolution between S and SCl_4_ during charge and discharge, confirming the feasibility of the high-valence sulfur redox chemistry (Fig. 1b). A high average Coulombic efficiency (CE) of ∼96% is realized in NaDCA-based Na–Al cells (Fig. 1c), much higher than that of NaCl-based Na–Al cells (20–50%). *In situ* optical imaging demonstrates highly uniform sodium deposition in the NaDCA electrolyte, whereas dendritic growth dominates in the NaCl system (Fig. 1d). The reported batteries also demonstrate remarkable electrochemical performance that is rarely realized in sulfur-based systems. Using a pristine sulfur cathode, the battery achieves a reversible capacity of 744 mAh g^−1^ (Fig. 1e), which could be further enhanced to 1206 mAh g^−1^ with the incorporation of 8 wt% bismuth-coordinated covalent organic framework (Bi-COF) catalyst. This corresponds to an electrode-level energy density exceeding 2000  Wh  kg^−1^ (based on the total mass of both cathode and anode). The fast reaction kinetics also supports excellent rate capability of up to 16  A g^−1^ and stable operation at a wide temperature range from −40 to 80°C (Fig. 1f). Moreover, the battery exhibits stable cycling performance during 1400 cycles at a charge capacity of 200 mAh g^−1^. These metrics underscore the effectiveness of the proposed high-valence sulfur chemistry in improving the long-standing performance of Na–S batteries.

Beyond the laboratory-scale performance, the authors also validate the potential of this Na–S battery in a practical scale. Safety tests, including ignition, cutting, and nail penetration, confirm the high safety provided by the non-flammable electrolyte. A scaled-up 1.06-Ah battery retains stable cycling behavior (Fig. 1g–i), suggesting the feasibility from laboratory-scale prototypes to commercial batteries. Owing to the use of abundant materials and simple architecture, the battery shows an estimated material cost of US$5.03 per kWh, making it highly attractive for grid energy storage (Fig. 1j). The high-valence sulfur redox chemistry is further extended to Li–S batteries, highlighting the broad applicability of the cathode reaction.

This work by Sun *et al.* pioneers high-valence alkali metal–sulfur batteries, introducing a transformative cathode chemistry enabled by synergistic materials and electrolyte engineering. By unlocking the S^0^/S^4+^ cathode chemistry and integrating an anode-free configuration, it challenges the long-standing reliance on low-valence sulfur chemistry and excess alkali metal. This advance is expected to stimulate renewed exploration of sulfur chemistries beyond conventional design boundaries. Future efforts should address the interfacial and chemical stability of chloroaluminate electrolytes under long-term high-voltage operation, particularly their compatibility with current collectors and reactive sulfur intermediates. More broadly, these interfacial processes may be also easily understood in terms of energy-state accessibility governing the transport of ions and electrons in a confined electrochemical environment [8]. Achieving reversible sodium plating under lean-electrolyte and high-areal-loading conditions will also be critical for practical deployment. Extending this high-valence sulfur chemistry to other alkali metals and scalable devices may further accelerate the development of sulfur batteries for grid storage and wearable applications.
